# Caregiving Intensity, Stress, and Social Relationships in Mexican American Dementia and Non-Dementia Caregivers

**DOI:** 10.1093/geroni/igaf122.1386

**Published:** 2025-12-31

**Authors:** Suyoung Kim, Anna Bokun, Sunshine Rote, Flavia Andrade, Phillip Cantu, Jacqueline Angel

**Affiliations:** The University of Chicago, Chicago, Illinois, United States; The University of Texas at Austin, Boston, Massachusetts, United States; University of Louisville, Louisville, Kentucky, United States; University of Illinois--Urbana-Champaign, Urbana, Illinois, United States; UTMB Health, The University of Texas Medical Branch, Galveston, Texas, United States; The University of Texas at Austin, Austin, Texas, United States

## Abstract

Mexican American caregivers often face intensive caregiving demands, which may be further complicated or protected by social and family dynamics. This study examines the mediating roles of social relationships, including support, negative interactions, and familism, in the relationship between caregiving intensity and perceived stress. Using the stress process paradigm, we assess whether these factors increase or reduce caregiver perceived stress and explore differences between dementia and non-dementia caregivers. Data from the Hispanic Established Populations for the Epidemiologic Study of the Elderly (Wave 9, N = 457) were analyzed using Structural Equation Modeling. Social support, negative interaction, familism, and perceived stress were modeled as latent constructs. A multiple-group SEM approach assessed differences in caregiving paths between dementia and non-dementia caregivers. Caregiving intensity was negatively associated with social support and familism but positively linked to negative interactions. Social support significantly buffered the relationship between caregiving intensity and perceived stress, whereas negative interactions exacerbated it. Dementia caregivers reported a stronger association between caregiving intensity and perceived stress than non-dementia caregivers. Social support helped reduce stress more for non-dementia caregivers, while negative interactions had a greater impact on dementia caregivers. High-intensity caregiving is associated with increased stress, particularly among dementia caregivers in Mexican American communities. Social relationships play a complex role, both alleviating and exacerbating caregiver burden. These findings underscore the need for interventions that strengthen support networks, reduce negative family dynamics, and address the unique challenges facing dementia caregivers, ultimately improving caregiver well-being and care sustainability.

